# Health and Functional Determinants of Orthostatic Hypotension in Geriatric Ward Patients: A Retrospective Cross Sectional Cohort Study

**DOI:** 10.1007/s12603-019-1201-2

**Published:** 2019-05-21

**Authors:** Zyta B. Wojszel, A. Kasiukiewicz, L. Magnuszewski

**Affiliations:** 1 Department of Geriatrics, Medical University of Bialystok, Fabryczna str. 27, 15-471, Bialystok, Poland; 2 Department of Geriatrics, Hospital of the Ministry of Interior in Bialystok, Bialystok, Poland; 3 Department of Geriatrics, Medical University of Bialystok, Bialystok, Poland

**Keywords:** Orthostatic hypotension, geriatric inpatients, frailty, comprehensive geriatric assessment, underestimation

## Abstract

**Objective:**

Orthostatic hypotension (OH) is a common problem in older people. Although it is indicated that OH can be a marker of frailty there are no studies that evaluate this relationship in hospitalized patients. The aim of the study was to assess the prevalence of OH in geriatric ward patients and its association with health and functional ability characteristics and patients' frailty status.

**Design and setting:**

A retrospective cross-sectional cohort study was conducted among patients aged 60 or over hospitalized in the geriatric ward.

**Participants:**

Patients' medical records were analyzed and those with Active Standing Test (AST) results were included in the study.

**Measurements:**

Orthostatic hypotension was defined by a drop in blood pressure of at least 20mmHg for systolic blood pressure and at least 10mmHg for diastolic blood pressure within 3minutes of standing up in AST. The database included sociodemographic characteristics, nutritional, functional and cognitive state, comorbidity and medical treatment. Frailty syndrome was diagnosed with Clinical Frailty Scale. Correlations with OH were counted and multivariable logistic regression models were built.

**Results:**

416 patients were hospitalized in the study period and 353 (84.9%) were included, 78 (22.1%) men and 298 (84.4%) 75+ year-old. AST was not available in patients significantly more dependent in ADL and more frail. OH was diagnosed in 57 (16.2%) patients, significantly more frequently in men (systolic- 45,5%, systolic-diastolic-40,0%). The significant independent predictors of OH were lower diastolic blood pressure at admittance, nutritional risk in MNA-SF, Parkinson disease, α1-blockers, neuroleptics and memantine, and not the frailty syndrome diagnosed with Clinical Frailty Scale.

**Conclusions:**

OH affects a significant percentage of patients in the geriatric ward, although this problem may be underestimated due to limitations in the performance of AST in very frail and functionally dependent patients.

## Introduction

Orthostatic hypotension (OH) is a common problem in older patients. It affects about 5–30% up to 80% of seniors, depending on the population studied as well as method and criteria of OH assessment used (1). Most studies of OH have been performed in population cohorts (2) or older residents of care facilities (3, 4, 5), but a problem of OH in hospitalized older patients was little studied to date (6, 7).

OH is closely associated with common chronic diseases, including hypertension, congestive heart failure, diabetes mellitus, and Parkinson's disease. Adverse drug reactions of drugs used in Parkinson's disease, hypertension, diabetic neuropathy, sleep disorders or depression may also lead to a sudden drop in blood pressure in upright position (8, 9, 10). It is also more common in individuals with atrial fibrillation (11).

Adverse consequences of OH independent of potential confounders include, among others, falls and fractures (7, 12, 13), poorer global cognitive function and poorer memory (14), increased risk of acute coronary syndrome, cardiac failure and stroke (15). Community-dwelling older adults with orthostatic hypotension have higher independent risk of developing new-onset heart failure, which appeared to be more pronounced in those with symptomatic orthostatic hypotension (16), and worse prognosis for long-term survival (17, 18, 19). Orthostatic hypotension-related admissions herald increased cardiovascular mortality (20). Some studies, however, indicate that this influences depend to a large extent on population characteristics in which OH's impact on prognosis is assessed. And so, for example, in a population of frail elderly patients who were assessed in the outpatient Comprehensive Geriatric Assessment Unit, with a high burden of comorbidity, OH was not an independent risk factor for overall mortality, whereas other factors such as degree of disease burden or chronic kidney disease played significant role. Therefore it is more likely that it could be treated rather as a marker of frailty that is easy to measure, and not as the independent risk factor for bad prognosis (21).

The aim of the study was to assess the prevalence and clinical determinants of OH in geriatric in-patients, with special regard to its correlation with functional ability characteristics and frailty status. To the authors' knowledge, there are no studies examining the relationship between OH and frailty syndrome in patients of geriatric wards.

## Participants and methods

We retrospectively analyzed medical records of all consecutive patients admitted to the geriatric ward of the Hospital of the Ministry of Interior in Bialystok, Poland, between 1st September, 2014 and 30th April, 2015 taking part in the prospective cross-sectional study devoted to different aspects of frailty syndrome in geriatric in-patients. The study population included patients with the Active Standing Test (AST) result documented in the medical record. AST is performed in the department usually in the morning on the second day of patient's hospital stay as a part of comprehensive geriatric assessment (CGA). After a resting period of 10 minutes, blood pressure is measured electronically (Automatic Philips IntelliVue MP5 Monitor) on the non-dominant arm with the patient lying down. The patient is then asked to stand up, and the blood pressure is measured again during the first and the third minute after standing (22). In the case when the patient cannot stand the measurement is made in a sitting position. The procedure is performed by a physiotherapist working in the geriatric department.

The following variables characterizing the patients were retrieved from medical charts: age, gender, blood pressure (BP) and pulse pressure (PP) records at admission, prevalence of diseases that can increase the risk of orthostatic hypotension, number of chronic diseases (of 15 diseases medically confirmed at discharge: peripheral arterial disease, ischemic heart disease, chronic cardiac failure, hypertension, stroke, atrial fibrillation, chronic obstructive pulmonary disease, diabetes/ prediabetes, neoplasm, thyroid gland disease, dementia, parkinsonism, chronic arthritis, chronic renal disease, dementia), number of medications taken at admission, and taking medicines that affect blood pressure and increase the risk of OH (included antihypertensive drugs: angiotensin converting enzyme inhibitors- ACE-Is/ angiotensin II receptor blockers- ARBs, beta-blockers, calcium channel blockers, diuretics, α1-blockers; digoxin; antiparkinsonian drugs; antidepressants: selective serotonin reuptake inhibitors- SSRI; antipsychotic treatment: in general and quetiapine separately; pro-cognitive medications: memantine, acetylcholinesterase inhibitors- ACHE-I). Physical and mental abilities of an older person were assessed, based on the results of tests and scales routinely carried out within comprehensive geriatric assessment performed in the geriatric ward (the ability to carry out basic activities of daily life with the Barthel Index (23), risk of pressure sores with Norton Scale (24), risk of recurrent falls with the Performance Oriented Mobility Assessment- POMA (25), and Timed Up and Go test-TUG (26), the cognitive state with the Abbreviated Mental Test Score-AMTS (27), the emotional state with 15 item Geriatric Depression Scale (28)). Hand grip strength of the dominant hand (mean of two measurements) was assessed using a manual hydraulic dynamometer SAEHAN DHD-1. Gait speed was measured during the 4.57m walk at usual pace. To measure the frailty, the 7 item Canadian Study of Health and Aging Clinical Frailty Scale (CFS) (29) was used. Information on nutritional status (risk of malnutrition with Mini Nutritional Assessment-Short Form- MNA-SF (30), body mass index- BMI, waist-hip ratio- WHR, calf circumference-CC, mid arm circumference-MAC, albumin level, hemoglobin level) and on renal function (glomerular filtration rate- GFR, counted using the CKD-EPI formula (31), serum creatinine level) was collected. Finally we also compared the characteristics of patients from the group in which AST was performed and in which this test was not performed.

### Study parameters

Orthostatic hypotension was defined by a drop in blood pressure of at least 20mmHg for systolic blood pressure and at least 10mmHg for diastolic blood pressure within 3minutes of standing up in AST. Frailty was defined as CFS score 5–7 and severe frailty — as CFS score of 6 or 7. High pulse pressure was defined as the difference between the systolic blood pressure and the diastolic blood pressure above 50mmHg at admittance to the department. Polypharmacy was defined as 5 or more drugs taken. Multimorbidity was defined as 5 or more diseases of 15 listed (as above). Chronic kidney disease (CKD) i.e. stage 3, 4 and 5 CKD according to Kidney Disease Outcome Quality Initiative (KDOQI) was diagnosed if GFR was &lt;60ml/min/1.73m2. The high risk of falls was diagnosed if POMA score was&lt;19, and if TUG time was ≥14s. Malnutrition was suspected if albumin level was &lt;35g/L, or if MNA-SF score was below 8. Sarcopenia was diagnosed if MAC was ≤22cm or CC was &lt;31cm. Slowness and weakness were diagnosed according to criteria proposed in the literature- cut off points were stratified by gender and height in case of slowness and by gender and BMI quartiles in case of weakness (32). The diagnosis of dementia at discharge was based on the thorough neuropsychological examination. Anemia was diagnosed if the hemoglobin level was below 8.69 mmol/L in men and below 7,45 mmol/L in women.

The study was approved by the Ethics Committee at Medical University in Bialystok (R-I-002/305/2013). All procedures performed in the study were in accordance with the ethical standards of the Medical University in Bialystok research committee and with the Helsinki declaration and its later amendments. Personal data were not identifiable during the analysis. The study can be classified as a study of ‘usual practice'. Data are available to researchers and applications should be made direct to the corresponding author.

### Statistical analysis

Data were collected and analyzed using IBM SPSS Version 18 Software suit (SPSS, Chicago, IL, USA), and presented as means and standard deviation for normally distributed and as medians and interquartile range for not normally distributed continuous variables, and the number of cases and percentage for categorical variables. Shapiro Wilk test was used to assess the distribution of variables. Proportions were compared using χ^2^ tests, while the independent samples t-test and Mann-Whitney U test were used to compare measures of central tendency (means and medians). It was followed by a multivariable logistic regression including all predictors with a P value less than 0.1. Missing values were omitted and statistics in such cases were calculated for the adequately reduced groups. A P value of less than 0.05 was regarded as significant.

## Results

There were 416 patients hospitalized in the study period and 353 (84.9%) had AST result documented in the medical record and were included in the study group. The median age of patients was 82 (77–86) and the majority of them was above 75 years of age (84.4%), and female (77.9%). OH was diagnosed in 57 (16.2%) patients, significantly more frequently in men. In 55 of patients with OH we managed to determine the type of hypotension. In the majority of cases (n=25; 45.5%) it was systolic-diastolic hypotension, less often it was systolic OH (n=22; 40.0%), and the least found was diastolic hypotension (n=8; 14.5%). In 61.8% of cases the orthostatic drop of blood pressure was noted only in the first minute of AST, in 32.7% in both measurements, and in 5.5% in the third minute of the test. In a large percentage of OH cases (44.4%), the assessment of orthostatic hypotension was possible in a sitting position only, as the patient was not able to keep a standing position.

To assess the homogeneity of the groups and evaluate the possible determinants of OH, we compared patients with OH (“OH+” group) and without OH (“OH−” group) according to AST (table 1- sociodemographic and functional characteristics; table 2- nutritional health characteristics and table 3- morbidity and pharmacological treatment). The groups did not differ in age, number of chronic diseases, and number of drugs taken. We found no significant correlation between OH and multimorbidity or polypharmacy. Barthel Index, AMTS, GDS, MNA-SF and Norton scale scores were similar in both groups. Gait speed, percentage of patients with slowness, POMA and TUG results, percentages of patients with high risk of falling according these tests and history of falls in the previous 12 months also did not differ between the groups. The prevalence of dementia, diabetes, chronic kidney disease, anemia, cardiovascular diseases (hypertension, ischemic heart disease, atrial fibrillation, peripheral arterial disease, history of stroke/TIA in the past) in both groups was similar. The usage of different classes of medications used in cardiovascular diseases (β-blockers, diuretics, ACE-Is/ ARBs, calcium channel blockers, digoxin) was also similar in both groups. The correlation between OH and AChE-Is and parkinsonian drugs (in the majority of cases levodopa) was also insignificant. The groups did not differ in median values of Clinical Frailty Scale and in the percentage of patients assessed as severely frail.

**Table 1:** Characteristics of the study groups- sociodemographic and functional correlates of OH

**Parameter**	**Total**	**OH+ group**	**OH− group**	**P value**^a^	**Missing values**
No. (%) of patients	353 (100.0)	57 (16.2)	296 (83.8)		
Age, Me (IQR)	82 (77–86)	82 (78–85)	82 (76.25–33.9)	0.73	-
Age, 75+, n (%)	298 (84.4)	49 (85.9)	249 (84.1)	0.73	-
Gender, men, n (%)	78 (22.1)	25 (43.9)	53 (17.9)	&lt;0.001	
Systolic BP, mmHg, Me (IQR)	130 (120–140)	125(112.5–133.5)	130 (120–140)	0.03	-
Diastolic BP, mmHg, Me (IQR)	70 (60–80)	70 (60–70)	70 (65–80)	0.001	-
PP, mmHg, Me (IQR)	60 (50–70)	60 (48.5–70)	60 (50–70)	0.99	-
PP&gt;50mmHg, n (%)	219 (62.0)	37 (64.9)	182 (61.5)	0.63	-
Barthel Index, Me (IQR)	90 (65–100)	85 (67.5–100)	95 (80–100)	0.35	-
AMTS, Me (IQR)	8.0 (6.0–9.0)	8.0 (6.0–9.0)	8.0 (6.0–9.0)	0.98	11
GDS score, Me (IQR)	6.5 (3.0–10.0)	6.0 (3.0–10.0)	7.0 (3.0–10.0)	0.94	25
Falls in the last year, n (%)	151 (46.60)	25 (49.02)	126 (46.15)	0.70	29
CFS, Me (IQR)	5.0 (4.0–5.0)	5.0 (4.0–5.5)	5.0 (5.0–5.0)	0.29	-
Severe frailty, n (%)	66 (18.7)	14 (24.6)	52 (17.6)	0.22	-
Norton scale score, Me (IQR)	18.0 (16.0–19.0)	17.0 (15.5–19.0)	18.0 (16.0–19.0)	0.18	-
Pressure sores risk, n (%)	38 (10.8)	7 (12.3)	31 (10.5)	0.69	
Gait speed, m/s, Me (IQR)	0.65 (0.40–0.93)	0.70 (0.37–0.90)	0.64 (0.40–0.95)	0.78	49
Slowness, n (%)	161 (52.96)	25 (50.0)	136 (53.54)	0.64	49
POMA, Me (IQR)	23.0 (18.0–28.0)	23.0 (17.0–28.0)	23.0 (18.0–28.0)	0.77	44
TUG, s, Me (IQR)	17.4 (12.0–28.0)	16.67 (12.63–25.88)	18.0 (11.59–28.0)	0.74	61


a. χ^2^ test or Fisher exact test, as appropriate, for categorical variables; Mann-Whitney test for interval variables; Abbreviations: AMTS- Abbreviated Mental Test Score; CFS- 7-point Clinical Frailty Scale; GDS- 15 items Geriatric Depression Scale; IQR- interquartile range; Me- median value; n- number of cases; OH- orthostatic hypotension; POMA- Performance Oriented Mobility Assessment; PP- pulse pressure; TUG- Timed Up and Go test.

**Table 2:** Characteristics of the study groups- nutritional correlates of OH

**Parameter**	**Total**	**OH+ group**	**OH-group**	**P value**^a^	**Missing values**
No. (%) of patients	353 (100.0)	57 (16.2)	296 (83.8)		
MNA-SF, Me (IQR)	12.0 (9.0–13.0)	12.0 (9.0–13.0)	18.0 (9.75–13.0)	0.53	8
MNA-SF score &lt;8, n (%)	49 (14.20)	13 (23.64)	36 (12.41)	0.03	8
BMI, kg/m^2^, M (SD)	29.25 (5.97)	28.10 (5.98)	29.60 (5.98)	0.10	-
BMI&lt;24 kg/m^2^, n (%)	59 (18.27)	10 (19.23)	49 (18.08)	0.84	30
BMI&gt;30 kg/m^2^, n (%)	140 (43,3)	16 (30.8)	124 (45.8)	0.04	30
WHR, Me (IQR)	0.91 (0.86–0.95)	0.91 (0.88–0.96)	0.9 (0.86–0.95)	0.28	-
MAC, cm, M (SD)	28.13 (3.96)	28.11 (4.18)	28.14 (3.92)	0.95	33
MAC≤22cm, n (%)	78 (22.09)	11 (22.00)	67 (24.81)	0.67	33
CC, cm, M (SD)	34.71 (4.48)	33.73 (4.59)	34.89 (4.44)	0.09	33
CC&lt;31cm, n (%)	56 (15.86)	13 (26.00)	43 (15,93)	0.09	33
Handgrip strength, kg, Me (IQR)	18.2 (13.85–22.75)	19.4 (15.5–23.3)	18 (13.6–22.7)	0.14	25
Weakness, n (%)	218 (66.46)	39 (70.91)	179 (65.57)	0.44	25
Albumin, g/L M (SD)	39.1 (3.7)	39.1 (3.7)	39.2 (3.7)	0.67	14


a - χ^2^ test or Fisher exact test, as appropriate, for categorical variables; Mann-Whitney test for interval variables. Abbreviations: BMI- body mass index; CC- calf circumference; IQR- interquartile range; M- mean value; MAC- mid-arm circumference; Me- median value; MNA-SF- Mini Nutritional Assessment-Short Form; n- number of cases; OH- orthostatic hypotension; SD- standard deviation; WHR- waist-hip ratio.

**Table 3:** Characteristics of the study groups- morbidity and pharmacological treatment

**Parameter**	**Total**	**OH+ group**	**OH−group**	**P value**^a^	**Missing values**
No. (%) of patients	353 (100.0)	57 (16.2)	296 (83.8)		
Number of chronic diseases^b^, Me (IQR)	5.0 (3.0–6.0)	5.0 (3.0–6.0)	5.0 (3.0–6.0)	0.29	-
Multimorbidity, n (%)	204 (57.8)	35 (61.4)	169 (57.1)	0.55	-
Parkinson disease, n (%)	39 (11.1)	11(19.3)	28 (9.5)	0.03	-
Dementia, n (%)	109 (30.9)	21 (36.8)	88 (29.7)	0.29	-
Depression, n (%)	160 (55.6)	25 (55.6)	135 (55.6)	1.0	65
Diabetes, n (%)	105 (29.7)	19 (33.3)	86 (29.1)	0.52	-
Chronic kidney disease -GFR&lt;60 ml/min/1.73m^2^, n (%)	193 (54.7)	31 (54.4)	162 (54.7)	0.96	9
Hypertension, n (%)	286 (81.0)	44 (77.2)	242 (81.8)	0.42	-
Ischemic heart disease, n (%)	199 (56.4)	31 (54.4)	168 (56.8)	0.74	-
Chronic cardiac failure, n (%)	134 (38.0)	22 (38.6)	112 (37.8)	0.91	
Atrial fibrillation, n (%)	77 (21.8)	12 (21.1)	65 (22.0)	0.88	-
Peripheral arterial disease, n (%)	55 (15.6)	12 (21.1)	43 (14.5)	0.21	-
Stroke/ TIA, n (%)	39 (11.1)	8 (14.0)	31 (10.5)	0.43	-
Anemia, n (%)	149 (42.8)	26 (45.6)	123 (42.3)	0.64	5
Number of drugs, Me (IQR)	7.0 (5.0-9.0)	8.0 (5.0–11.0)	7.0 (5.0–9.0)	0.27	8
Polypharmacy, n (%)	278 (80.6)	44 (80.0)	234 (80.7)	0.99	8
α1-blockers, n (%)	25 (7.3)	8 (14.6)	17 (59)	0.02	9
Neuroleptics, n (%)	55 (16.0)	15 (27.3)	40 (13.84)	0.01	9
Quetiapine, n (%)	41 (11.9)	13 (23.6)	28 (9.7)	0.003	9
AChE-I n (%)	36 (10.5)	7 (12.7)	29 (10.0%)	0.55	9
Memantine, n (%)	12 (3.49)	5 (9.09)	7 (2.42)	0.01	9
ACE-Is/ARBs, n (%)	224 (65.10)	32 (58.2)	192 (66.4)	0.24	9
ß-blockers, n (%)	219 (63.66)	34 (61.82)	185 (64.01)	0.76	9
Calcium channel blockers, n (%)	102 (29.7)	17 (30.9)	85 (29.4)	0.82	9
Digoxin, n (%)	25 (7.27)	2 (3.64)	23 (7.96)	0.26	9
Diuretics, n (%)	170 (49.4)	30 (54.5)	140 (48.4)	0.40	9
SSRI, n (%)	98 (28.49)	20 (3.36)	78 (26.99)	0.16	9
Antiparkinsonian drugs, n (%)	29 (8.2)	7 (12.3)	22 (7.4)	0.22	2


a - χ^2^ test or Fisher exact test, as appropriate, for categorical variables; Mann-Whitney test for interval variables; b. of 15 chronic diseases (peripheral arterial disease, ischemic heart disease, chronic cardiac failure, hypertension, stroke, atrial fibrillation, chronic obstructive pulmonary disease, diabetes/ prediabetes, neoplasm, thyroid gland disease, dementia, parkinsonism, chronic arthritis, chronic renal disease, dementia); Abbreviations: ACEIs- angiotensin converting enzyme inhibitors; AChE-I- acetylcholine esterase inhibitors; ARBs-angiotensin II receptor blockers; GFR- glomerular filtration rate; IQR- interquartile range; M- mean value; Me- median value; n- number of cases; OH- orthostatic hypotension; TIA-transient ischemic attack; SD- standard deviation; SSRI- selective serotonin reuptake inhibitors.

OH+ group and OH− group differed significantly in gender-OH was observed significantly more frequently in men. Median values of systolic and diastolic conventional blood pressure measured at admission were significantly lower in OH+ group, but the prevalence of the high pulse pressure was similar in both groups. The groups differed significantly in the percentages of patients at risk of malnutrition, i.e. with MNA-SF score&lt;8, but BMI, WHR, mean albumin and hemoglobin levels were similar in OH+ and OH− groups, whereas BMI &gt;30 kg/m2 was observed significantly less frequently in OH+ group. Handgrip strength, percentage of patients diagnosed with weakness, and MAC circumferences did not correlated with OH, but the association between lower CC circumferences suggesting sarcopenia and OH were on the verge of significance (p&lt;0.1). Percentage of patients with Parkinson disease was significantly higher in the OH+ group. Patients in OH+ group were also significantly more frequently taking α1-blockers, neuroleptics (especially quetiapine) and memantine.

Variables meeting the criterion p &lt;0.1 were assumed to be included in the regression analysis. Among these variables mail gender and use alfa1-blockers were strongly correlated with each other- alfa1-blockers were not reported at all in women. Therefore, although this variable was not connected with the significant multicollinearity effect, we decided to check two models of determinants of OH− the model including variable “gender” and not including it one.

A direct logistic regression analysis was performed on OH as outcome and 10 attitudinal predictors: gender (men), systolic and diastolic blood pressure values, risk of malnutrition (MNA-SF score &lt;8), BMI&gt;30 kg/m2, calf circumference (CC), Parkinson disease, taking neuroleptics, memantine, and α1-blockers. An independent positive effect associated with the prevalence of OH in AST was observed among male patients, with lower values of diastolic blood pressure, at risk of malnutrition according to MNA-SF, and taking memantine. In the model without variable “gender” Parkinson disease and taking alfa1-blockers revealed to be significant independent predictors of OH apart from previously mentioned ones.

Because the AST- albeit an obligatory part of a comprehensive geriatric assessment procedure in the department- was not reported in medical records of quite a large (15.1%) percentage of patients, we looked at the differences between patients with AST and without this test results. The groups did not differ in age, gender and in a number of characteristics, taken into account in previous analyzes, but they differed in terms of degree of disability in ADL and I-ADL. Significantly more frequently AST was not made in more frail people, with higher scores of Clinical Frailty Scale and classified as severely frail (table 5).

**Table 5:** Characteristics of the groups with AST performed (AST+) and not performed (AST−)

**Parameter**	**AST+**	**AST−**	**P value**^a^
No. (%) of patients	353 (84.9)	63 (15.1)	
Age, Me (IQR)	82 (77–86)	83 (77–87)	0.38
Age, 75+, n (%)	295 (84.4)	52 (82.5)	0.71
Gender, men, n (%)	78 (22.1)	16 (25.4)	0.56
Barthel Index, Me (IQR)	95 (80–100)	35 (10–86.25)	&lt;0.001
IADL, Me (IQR)	8 (4–11)	0 (0–7.5)	&lt;0.001
CFS, Me (IQR)	5 (4–5)	6 (4–7)	&lt;0.001
Frailty, n (%)	186 (52.7)	44 (69.8)	0.01
Severe frailty, n (%)	66 (18.7)	36 (57.1)	&lt;0.001


a. χ^2^ test for categorical variables; Mann-Whitney test for continuous or interval variables; Abbreviations: CFS- 7-items Clinical Frailty Scale; IADL- instrumental activities of daily living; IQR- interquartile range; Me- median value; n- number of cases.

## Discussion

The results of our study revealed that OH affects a significant percentage of patients in the geriatric ward. It was observed in above 16% of patients tested for OH. On the other hand the AST was not performed in almost similar (15.1%) percentage of patients, mainly severely frail and dependent in ADL, and in quite a high percentage of patients the orthostatic hypotension was diagnosed in a sitting position as they were not able to keep standing. It may indicate that due to limitations in the performance of AST, the problem of OH may be underestimated in very frail and disabled patients' population. Also a number of other factors may affect the underestimation of the incidence of orthostatic hypotension in geriatric patients in daily clinical practice. In older people it is recommended to evaluate towards the occurrence of OH regardless the presence of postural symptoms. A retrospective analysis of outpatients consequently afferent to a tertiary hospital in Milan for memory complaints, functional evaluations or comprehensive geriatric assessments during the years 2011–2014 revealed that only in 3% of them OH was found, although a high percentage of them had symptoms suggestive for OH (33). Considering the main characteristics of the patients in this Italian study (very similar to our study group) it is difficult to think that OH prevalence could really be so low, but it can rather suggest- as it was proposed by the authors of the study- the malpractice and not adhering to the gold standard recommendations for the blood pressure measurement in the clinical setting (34). We can suspect that to some extent it may also relate to the participants in our study, albeit AST should be an obligatory part of a comprehensive geriatric assessment procedure in the department.

**Table 4:** Risk factors associated with OH− multivariable logistic regression models

	**OR**	**95% CI MODEL 1**	**P value**	**OR**	**95% CI MODEL 2**	**P value**
Gender (men)	3.55	1.4–8.7	0.006			
Systolic blood pressure	1.002	0.98–1.0	0.90	1.003	0.98–1.03	0.81
Diastolic blood pressure	0.95	0.9–0.99	0.01	0.94	0.90–0.98	0.01
MNA-SF score &lt;8	2.8	1.0–7.6	0.047	2.69	1.01–7.15	0.047
BMI&gt;30 kg/m^2^	0.6	0.2–1.6	0.32	0.55	0.20–1.46	0.23
CC	1.1	0.95–1.2	0.25	1.07	0.96–1.21	0.23
Parkinson disease	2.6	0.96–6.9	0.06	3.16	1.23–8.13	0.02
Neuroleptics	1.7	0.69–3.97	0.26	1.88	0.80–4.41	0.15
Memantine	5.2	1.1–23.5	0.03	4.91	1.15–20.98	0.03
α1-blockers	1.3	0.39–4.34	0.67	3.49	1.26–9.68	0.02


Abbreviations: BMI- body mass index; CC- calf circumference; CI- confidence interval; MNA-SF- Mini Nutritional Assessment-Short Form; OR- odds ratio.

OH prevalence may also differ depending on the population studied as well as method and criteria of OH assessment used- AST or, for example, beat-to-beat technology (35, 36). The pooled prevalence of OH in community-dwelling older people (over 60 years) was 22.2% and 23.9% in long-term settings (36). In a random sample of persons aged 75 years or older in the city of Kuopio, Finland OH prevalence was 34% (37), similarly to its prevalence among home care clients aged 75 years or older living in Eastern and Central Finland- 35.7% (38), whereas the frequency of symptomatic OH at the Parkinson's Disease Center and Movement Disorders Clinic, Baylor College of Medicine, was as high as 81% in patients with multiple system atrophy (MSA), and only 18% of PD patients, and 19% of patients with non-MSA atypical parkinsonism (39), and patients with OH were significantly older.

Studies have not always shown a relationship between OH and age. Although OH was observed more frequently with age in some studies (39, 40), in others the age itself did not turn out to be an independent risk factor for OH in multivariate analyzes (41). In our study we did not observe any correlation between OH and the advancement of age, but the study group was not a randomly chosen sample from the general population. It was constituted of patients consecutively admitted to the geriatric department, more ill and frail then the average of community dwelling older people.

Data from studies regarding correlation between OH and gender are contradictory. In some studies gender was not connected with the prevalence of OH (37), but in our study OH was observed significantly more frequently in men. It is in accordance with observations that female subjects were better able to maintain cerebral flow velocities during postural changes and demonstrated better cerebral autoregulation, although the mechanisms of sex-based differences in autoregulation remained unclear (42). But in some studies the opposite correlation was observed- female sex was associated with larger declines in BP upon standing (43). Based on our study results we relate gender difference rather not with sex itself but with the specificity of health problems and their therapies in women and men. First of all alfa1 receptor blockers were used only by men in our group, and it had been proved for them that they were a strong predictor of OH in many studies (41, 44). Similarly neuroleptics were reported at admittance also significantly more frequently in men in our study (data not presented), and they were also connected with the higher risk of OH in some studies (35).

According to some authors OH may be treated as a marker of clinical frailty (21) but we did not found this kind of direct correlation in our study. Clinical frailty scale results did not correspond with the prevalence of OH. One of the reasons can be generally high prevalence of frailty (55,2% of study population) and co-occurrence of different geriatric problems in this group as the whole. It can be also the result of the fact that in the most frail and functionally dependent patients the AST test was not performed at all. On the other hand the MNA-SF score &lt;8 suggesting risk of malnutrition turned out to be one of the main independent predictors of OH. Lower mean MNA scores appeared to be independently connected to a risk of OH also in Luukkonen et al. study (38). This may nevertheless suggest a relationship between the frailty status and OH.

Contrary to other studies' results we did not observe any specific associations between orthostatic hypotension and multimorbidity, polypharmacy, prevalence of cardiovascular diseases, or different classes of cardiovascular medications use. But the median values of systolic and diastolic conventional blood pressure measured at admission were significantly lower in OH+ group in our study, and lower diastolic blood pressure was one of the main independent predictors of OH in logistic regression. Low blood pressure on admission to the ward may have resulted, at least in some of these patients, from an inadequate hydration status- a frequent problem in geriatrics. But it could also be a result of too intensive antihypertensive therapy. Until the results of HYVET Study it was considered both appropriate and necessary to treat elderly hypertensive patients aggressively to the same target blood pressures identified for younger patients because a number of clinical trials demonstrated that treatment of hypertension significantly reduces the cardiovascular event rate in older patients (45). But in patients with multiple comorbidities and/or extensive atherosclerotic burden the J curve exists, and the benefits of reducing systolic or diastolic blood pressure to low values may be dangerous, leading even to an increase in total mortality (46). It was confirmed that discontinuation of antihypertensive medication may increase the probability of recovery from orthostatic hypotension in older patients (47). In our study the only group of cardiovascular medications independently predicting the prevalence of OH was alfa1-blockers. The risk of OH connected with this medications is well established (41, 44, 48), and was also confirmed by our study results.

Parkinson's disease was one of the main independent predictors of OH in our study. It corresponds with the results of other studies (49). This correlation may be connected with so called neurogenic orthostatic hypotension, which prevalence ranges from 40 to 60% in Parkinson disease throughout the course of the disease. It results from a failure of the autonomic nervous system to regulate blood pressure in response to postural changes due to an inadequate release of norepinephrine, leading to supine hypertension and orthostatic hypotension (50). Medications used in Parkinson's disease also lower blood pressure, although we failed to demonstrate the relationship between OH and parkinsonian medications use in our study.

Dementia was proved to be independently associated with OH in the older adult population in some studies (40), and OH may be blamed of almost half of syncope events in dementia patients (13). The scientific literature suggests that the relationship between cardiovascular autonomic dysfunction and dementia can be a bilateral one- the more serious dementia, the higher the risk of OH, but OH can also negatively affect the cognitive ability. We did not observe a direct correlation between OH and dementia, but the use of memantine, one of the pro-cognitive medications recommended in moderate and severe dementia, was one of the significant independent predictors of OH in our study. The use of this drug is usually associated with the risk of increased blood pressure, but incidences of orthostatic blood pressure drop are also reported (51, 52). Numerous psychiatric medications (such as neuroleptics, SSRIs, benzodiazepines) are well-known triggers of clinically relevant blood pressure changes (51). Psychotropic drugs were proved to be an important risk factor of OH in older people (35), and it was also confirmed in our study. We did not observe such a connection with SSRIs' use, observed by other researchers (41).

We did not observe any correlation between OH and variables describing risk of falling (such as gait speed, TUG or POMA tests results) or history of falls in the last 12 months. It may be connected with the retrospective study scheme and no assessment of the occurrence of OH available in the most frail patients. The recent individual patient data (IPD) meta-analysis of prospective observational studies showed a clear and significant relationship between OH and time to the first fall incident (7). By preventive measures, diagnosing orthostatic hypotension early enough and implementing proper treatment, one can reduce the occurrence of adverse events such as falls and fractures, and thus minimize the risk of decreasing geriatric patients' quality of life (53).

The results of our study have to be interpreted with caution, as the study has got some limitations, which should be mentioned. First of all, it was performed not in the sample randomly selected from the general population of older people, so the results can be generalized for the patients of the similar settings only. As it was a partially retrospective study so some limitations can result from its retrospective design (ie, data on some of the variables were not available for all the patients, as indicated in tables). However, we were striving to meet the requirements of a prospective observation (i.e., all consecutive patients with AST test result admitted to our department in the study period were included).

## Conclusions

OH affects a significant percentage of patients in the geriatric ward, although this problem may be underestimated due to limitations in the performance of AST in very frail patients. Diastolic hypotension on admission to the ward, malnutrition risk according to MNA-SF, Parkinson's disease and some drugs used (alfa1-blockers, neuroleptics and memantine), and no frailty status per se, were significant predictors of OH.
